# Alterations of acid-base- and electrolyte status immediately following electroconvulsive therapy

**DOI:** 10.1007/s00702-026-03110-6

**Published:** 2026-01-31

**Authors:** C. Stephani, V. Wentzler, D. Zilles-Wegner

**Affiliations:** 1 Department of Anesthesiology, University Medical Center Göttingen, Robert-Koch-Straße 40, 37073 Göttingen, Germany; 2 Department of Neurology, University Medical Center Göttingen, Robert Koch-Str. 40, 37075 Göttingen, Germany; 3 Department of Geriatric Medicine - Subsection Neurology, St. Martini Hospital, Göttinger Straße 34, 37115 Duderstadt, Germany; 4 Department of Psychiatry, University Medical Center Berlin - Charité, Berlin, Germany; 5 Department of Psychiatry and Psychotherapy, University Medical Center Göttingen, Von-Siebold-Str. 5, 37075 Göttingen, Germany

**Keywords:** Electroconvulsive therapy, seizures, blood-gas analyses, acid-base status

## Abstract

Electroconvulsive therapy (ECT) is an established treatment for severe affective and psychotic disorders. However, there are only few studies on metabolic short-term changes, for example in acid-base balance and electrolyte concentrations after ECT. In this study, serial venous blood gas analysis was utilized to systematically record alterations in these parameters and evaluate them in relation to safety-relevant aspects. Blood samples were obtained via a peripheral intravenous catheter immediately, and then 5, 15, 30, and 60 min after ECT in patients requiring either first or repeat ECT. Blood-gas analyses were performed to monitor concentrations of electrolytes and acid-base status among others. A repeated measures analysis of variance was applied to test for longitudinal changes of blood gas parameters. 47 ECT sessions were included in the final data analysis (19 patients, mean age 54.4 ± 12.7 years, 12 females). Following ECT, there were significant changes in electrolytes and acid-base parameters. The most significant effects were observed in the acid-base status, where there was decline in pH (reference range 7.35–7.45) from a mean of 7.39 to 7.29 followed by a recovery to near normal values within one hour (F(3.4;156.4) = 105.5; *p* < 0.001; *n* = 47). Except of an increase in calcium, there were no relevant changes in electrolyte levels. This study demonstrates a statistically significant drop of serum pH after ECT based on complementary alterations in lactate and carbon-dioxide levels. Most of these alterations may simply be due to the convulsive exertion of the tourniquet region. However, they are short-lived and return to baseline within one hour confirming the safety of the method with respect to acid-base alterations.

*Clinical Trial Registration* This study was prospectively registered on April 20, 2020 in the German trial register (https://www.drks.de/drks_web/) The trial registration number is DRKS00021467.

## Introduction

Electroconvulsive therapy is a first line treatment for a range of acute psychiatric illnesses. Its efficacy relies on the induction of an epileptic seizure under controlled conditions. Seizures induce a range of metabolic changes, that have been particularly investigated in the context of spontaneous epileptic seizures in patients with epilepsy (Rao et al. 1989). (Likewise, due to muscle fiber exertion, convulsive seizures often lead to a significant increase of creatine-kinase, which in rare cases may induce renal failure (Mishra and Dave 2013; Willert et al. 2004). Relatively few studies have investigated the changes in acid-base status following epileptic seizures. In one of them, serial blood samples had been taken from eight patients after they had had a tonic-clonic seizure. They demonstrated significant lactic acidosis (lactate concentration 12.7 ± 1 mmol/l, pH 7.14 ± 0.06) immediately after the seizure with clear improvement and normalization within one hour, respectively (lactate 6.6 ± 0.7 mmol/l; pH 7.38 ± 0.04) (Orringer et al. 1977). In a more recent study blood samples were taken from 32 adult patients with epilepsy approximately 10 min and 2, 6, 24 and 48 h after tonic-clonic seizures recorded on a video EEG monitoring unit (Nass et al. 2019). Serum lactate levels increased significantly in approximately 90% of seizures, by an average of 8.7 times the baseline value, while ammonia levels increased after approximately 70% of seizures and by an average of 2.6 times the baseline value. Both levels returned to normal after two hours. Blood levels of calcium, magnesium and sodium exhibited a slight increase, yet generally returned to baseline within a two-hour period. In contrast, potassium and chloride levels remained unaltered. Furthermore, an immediate increase in Glucose levels was observed following epileptic seizures with an average increase of 14% compared to baseline. Bakes et al. (2011) selected 100 patients admitted by emergency physicians for loss of consciousness. Of these patients, 50 were diagnosed with a generalized epileptic seizure, while the remaining 50 constituted the control group and were diagnosed with syncope. In the postictal group, the median bicarbonate level was significantly reduced (median at 17 [14–34 mmol/l] [reference range 22–26 mmol/l]), whereas the bicarbonate level was within normal limits after syncope (23 mmol/l [20–24 mmol/l].

There are few data regarding such alterations in the context of seizures induced by electroconvulsive therapy (ECT) and often, studies have been conducted during periods with more restricted technical capabilities. For example, in patients undergoing ECT venous CO2 partial pressure increased from 30 after hyperventilation and at the begin of the ECT to 38 mmHg during the seizure phase and then returned to the baseline value of 30 mmHg (Szirmai et al. 1975). There was only a minor change of the pH of peripheral blood towards acidosis, whereas a slight shift towards alkalosis was observed in the venous blood of the internal jugular vein. However, the pH always remained between 7.4 and 7.5 (Szirmai et al. 1975). With regard to lactate acidosis, Eiduson et al. measured a 6-fold increase in the initial lactate level after ECT without muscle relaxants (1953), i.e. after complete tonic-clonic seizures, which is comparable to the aforementioned results in patients with epilepsy (Nass et al. 2019). While the observations of Eiduson et al. (1953) have been obtained in patients that did not receive sedation and muscle relxataion prior to introduction of ECT (Eiduson et al. 1953). However, other studies have shown that the lactate value also may increase after administration of a muscle relaxant following ECT, which may be due to low doses of non-weight-adjusted succinylcholine (Mitis et al. 1954). Conversely, there are some typical well-documented systemic responses to ECT, such as an initial parasympathetic response that lasts only a few seconds and is accompanied by bradycardia or asystole, followed by activation of the sympathetic response after 5–15 s, leading to an increase in cardiac output, heart rate, cerebral blood flow and intracranial pressure (Wagner et al. 2005). In addition to adrenaline, vasopressin, adrenocorticotropic hormone, prolactin, cortisol and oxytocin (Aminoff et al. 1984; Meierkord et al. 1994) metabolites such as lactate and muscle enzymes are released during seizures as indicated before. The increase of the latter depends on the technique of muscle relaxation (Lipka and Bülow 2003; Matz et al. 2018). Lactic acidosis is transient and compensated by various metabolic processes and respiration trying to maintain pH within physiologic limits as described by the Henderson-Hasselbalch-equation, which describes the relation of acids and bases in a buffer-system with the pH (Orringer et al. 1977; Lipka and Bülow 2003). Nonetheless, acidosis is associated with reduced contractile cardiac function, reduced stroke volume, and increased resistance of the pulmonary arteries, depending on the type of acidosis (metabolic, respiratory) (Stengl et al. 2013). The severity of these effects correlates with the severity of the acidosis and therefore may put patients with preexisting cardiac dysfunctions at risk (Rodríguez-Villar et al. 2021).

Given the options provided by modern instant metabolic diagnostic tools and the relative scarcity of the corresponding scientific literature, we aimed at profiling metabolic changes detectable by blood gas analysis in the first hour after ECT and (1) compare these results with those published on spontaneous epileptic seizures and (2) explore the extent and clinical relevance of metabolic changes.

## Methods

The present study was approved by the Ethics Committee of the University Medical Center Göttingen (Ethics Application No. 33/1/17). Patients were recruited from the Department of Psychiatry and Psychotherapy of the University Medical Center Göttingen and their eligibility for the study was assessed according to the inclusion and exclusion criteria listed below. Patients were included in the study only after obtaining their informed consent.

### Study cohort

Adult patients with clinical indication for acute or maintenance ECT were able to participate in this study. Hence, patients were recruited without preferences for specific diagnoses. Exclusion criteria were lack of capacity to consent, relevant neurological (e.g. later stage neurodegenerative disease, neuroinflammatory diseases, history of ischemic stroke) or other systemic illness (e.g. severe hemato-oncological illness, relevant cardiac insufficiency, renal failure), organic affective disorder, substance dependence other than tobacco/nicotine, and dementia.

### Study design and procedures

The present study was an open, observational study with the aim of describing the course of blood gas analysis (BGA) parameters in the first hour immediately after electroconvulsive therapy in relation to the respective baseline values. Blood sampling and immediately consecutive BGA was performed in an on-site blood gas analyser a few minutes before starting preoxygenation, anesthesia and ECT, immediately after and 5, 15, 30 and 60 min after ECT. There was no specific hyperventilation procedure prior to anesthesia. The investigations were always integrated into the clinical routine of ECT application, as described below: In addition to basic telemetry, patients received a peripheral intravenous cannula (Vasofix^®^ Safety 1.10 × 33 mm G 20 pink or 0.90 × 25 mm G 22 blue) required for anaesthesia, followed immediately by blood sampling for the baseline measurement. A complete electrolyte solution was then added as a base infusion in a 500 ml electrolyte batch bottle (Sterofundin^®^ ISO, Ecoflac^®^ plus 500 ml, B. Braun, Melsungen). On average, one infusion bottle was used per treatment window (approximately 90 min).

ECT was performed with a Thymatron IV device (Somatics, LLC., Lake Bluff, IL, USA). The double-dose program and brief pulse technique were utilised, with the maximum dose set at 1008 mC and a pulse width ranging from 0.5 to 1.0 ms. Age method was used for dosing, i.e., patients were treated with ‘stimulus dose in % = age’ in case of right unilateral electrode placement and ‘stimulus dose in % = age * 0,5’ in case of bilateral electrode placement. Stimulus dose and electrode placement could be adapted when necessary to ensure adequate seizure quality, effectiveness and tolerability. Anaesthesia was administered in accordance with a predetermined protocol, the specific parameters of which were determined by the anaesthesiologists. Propofol and S-ketamine were administered in weight-adjusted doses as hypnotics, and glycopyrronium-bromide was added as an anti-salivatory agent. Dexamethasone (4 mg) was administered when the medical history was positive for post-anesthetic nausea. The cuff method was applied on the upper arm of one side and utilised to ascertain the motor seizure, while the Thymatron EEG was employed to determine the duration of the EEG seizure. The blood samples required for the study were obtained from the intravenous catheter (Vasofix Safety^®^, B. Braun, Melsungen, Germany) that had been placed immediately prior to this. For this purpose, it was necessary to briefly congest the blood above the intravenous cannula. In order to obtain non-diluted blood, the initial 5 ml of the aspirate were then discarded and the final 1–2 ml collected for analysis in a blood gas analyser (Gem Premier 4000^®^, Werfen, Munich) at six predefined time points, including measurements of electrolytes: Sodium (Na+), potassium (K+), calcium (Ca2+), chloride (Cl-); blood gases: Carbon dioxide (CO2) and Oxygen (O2) (as partial pressures (pCO2 and pO2)); pH (as well as derived or calculated values, namely the standard bicarbonate ion concentration (HCO3- (std)) as well as current and standard base deviation (base excess); base excess is a calculated parameter that indicates the status and equilibrium in the acid-base system. Practically, it is defined as the amount of buffer that is required to normalize the pH of an amount of extracellular fluid to pH 7.4 under standardized conditions (pCO_2_ = 40 mmHg, temperature = 37.0 °C). It requires measurement of pH and HCO3- and is calculated by applying a specific formula, e.g. the formula of van Silke being: BE = (HCO3- −24.4) + (2.3 * Hb + 7.7) * (pH-7.4) * (1-0.023.023 * Hb). Others: glucose, hemoglobin and lactate.

### Statistics

 Means and standard deviations were calculated for age, weight, and height, as well as vital signs, stimulation dose, motor seizure duration, EEG seizure duration, and medication doses used. A general linear model with repeated measures and a significance level of α = 0.05 and an intersubject factor of sex was used for each laboratory parameter. The sphericity of the distribution of the dependent variables was tested using the Mauchly test. In the absence of sphericity, the degrees of freedom were adjusted according to Greenhouse-Geisser. *Post-hoc* tests were applied to compare each time point of the study when the linear model with repeated measurements indicated a significant change in the parameter of interest over time. The Bonferroni method was used to prevent the accumulation of alpha errors. In the case of missing values in the measurement series, imputation procedures were used. In the case of missing values with a preceding and subsequent measurement, the mean of the two neighboring measurements was calculated. If a final value was missing, the mean of all previous measurements was calculated and used. Correlation analyses were performed by calculating Pearson’s correlation index with a predetermined significance level of < 0.05 to detect possible correlations between the maximum difference of a laboratory parameter from baseline and patient height, weight, age, motor-, and EEG seizure duration. We used Excel^®^ (Microsoft Office) and SPSS 28^®^ (IBM) software.

## Results

47 ECT sessions of 19 patients (12 female (70.2%); 54.4 ± 12.7 (19–77) years of age, 171 ± 7.5 cm, 80 ± 14.4 kg) were included in the study, resulting in 282 blood samples, which were analyzed with a blood gas analyzer. The main diagnoses of the patients were major depression (single episode F32.2 (*n* = 9), recurrent episodes F33.2 (*n* = 8)) in ICD-10 codes), bipolar disorder with current major depressive episode (F31.4 (*n* = 1)), and schizoaffective disorder with current depression (F25 (*n* = 1)). Patients received propofol (40 ± 9 mg per patient), S-ketamine (54 ± 12 mg per patient) and succinylcholine (94 ± 18 mg per patient). In addition, patients received glycopyrronium bromide 0.2 mg/ml to reduce salivation, 4 mg dexamethasone as antiemetic (in 6 cases), and 2–2.5 g metamizol as analgesic co-therapy. Patient characteristics and medications are summarized in Table 1.

**Table 1:** Patient demographics, main diagnoses and medications

	Sex	Age	cm	kg	ICD10	ECTs	Medication
P1	♀	51	175	75	F33.2	1	Sertraline, mirtazapine, risperidone, lorazepam
P2	♀	62	167	69	F32.2	5	Setraline, quetiapine, l-thyroxine, pantoprazole
P3	♀	53	162	69	F32.2	3	Quetiapine, lithiume, l-thyroxine, estradiole
P4	♂	59	180	100	F32.2	3	Duloxetine, l-thyroxine, pantoprazole, tamsulosine, finasteride, irbesartane, atorvastatine,
P5	♂	52	168	86	F33.2	2	Venlafaxine, trimipramine, quetiapine, apixaban, ramiprile, amlodipine, moxonidine, simvastatine, l-thyroxine, dapagliflozine, metformine, sitagliptine, dihydrotachysterole
P6	♀	39	169	73	F31.4	5	Prothipendyle, aripripazole, naltrexone, sertraline, olanzapine, lithiume
P7	♀	59	175	74	F25.1	2	Venlafaxine, olanzapine
P8	♀	19	167	59	F32.2	1	Venlafaxine, risperidone, diazepame, desogestrel
P9	♂	65	179	85	F32.2	3	Protipendyle, zolpidem, simvastatine, hydrochlorothiazide, sitagliptine, olmesartane, lorazepame, venlafaxine, acetyl-salicylic acid
P10	♀	66	165	80	F33.2	2	Venlafaxine, lithiume, mirtazapine, quetiapine, bisacodyl
P11	♀	54	174	66	F33.2	2	Moclobemide, lithiume, quetiapine, pantoprazole
P12	♀	59	164	62	F33.2	7	Lithiume, mirtazapine, venlafaxine, quetiapine, irbesartane
P13	♀	57	159	67	F32.2	1	Amitriptyline, zopiclone, pantoprazole
P14	♂	55	188	103	F33.2	3	Amitriptyline, duloxetine, zopiclone, haloperidole, aripiprazole, lorazepame, tamsulosine, pravastatine
P15	♂	47	174	83	F32.2	1	Fluoxetine, quetiapine, alprazolame
P16	♀	36	170	90	F32.2	1	Trimipramine, risperidone, propranolole, l-thyroxine, pantoprazole
P17	♂	59	177	107	F33.2	2	Lithiume, bupropione, quetiapine, ramiprile, pantoprazole
P18	♀	65	162	94	F32.2	2	Mirtazapine, sertraline
P19	♂	77	–	–	F32.2	1	–

Cm refers to the patients’ height, kg to their weight. The column headed “ECTs” indicates how many ECT sessions of the respective patient were included in this study

27 sessions were part of a first and 20 sessions part of a maintenance ECT series. Bitemporal (BT) electrode placement was used in 4 cases (8.5%), right unilateral (RUL) placement in 11 (23.4%) and left anterior right temporal configuration (LART) in 32 stimulations (68.1%). The average stimulus dose was 69 ± 37%. The average motor seizure duration was 31 ± 14 s, the average EEG seizure duration was 46 ± 21 s. Imputations due to missing data were applied for pH (six values), lactate (one value), sodium (14 values), and calcium (15 values).

### Changes of acid-base and blood-gas homeostasis

**Fig. 1:**
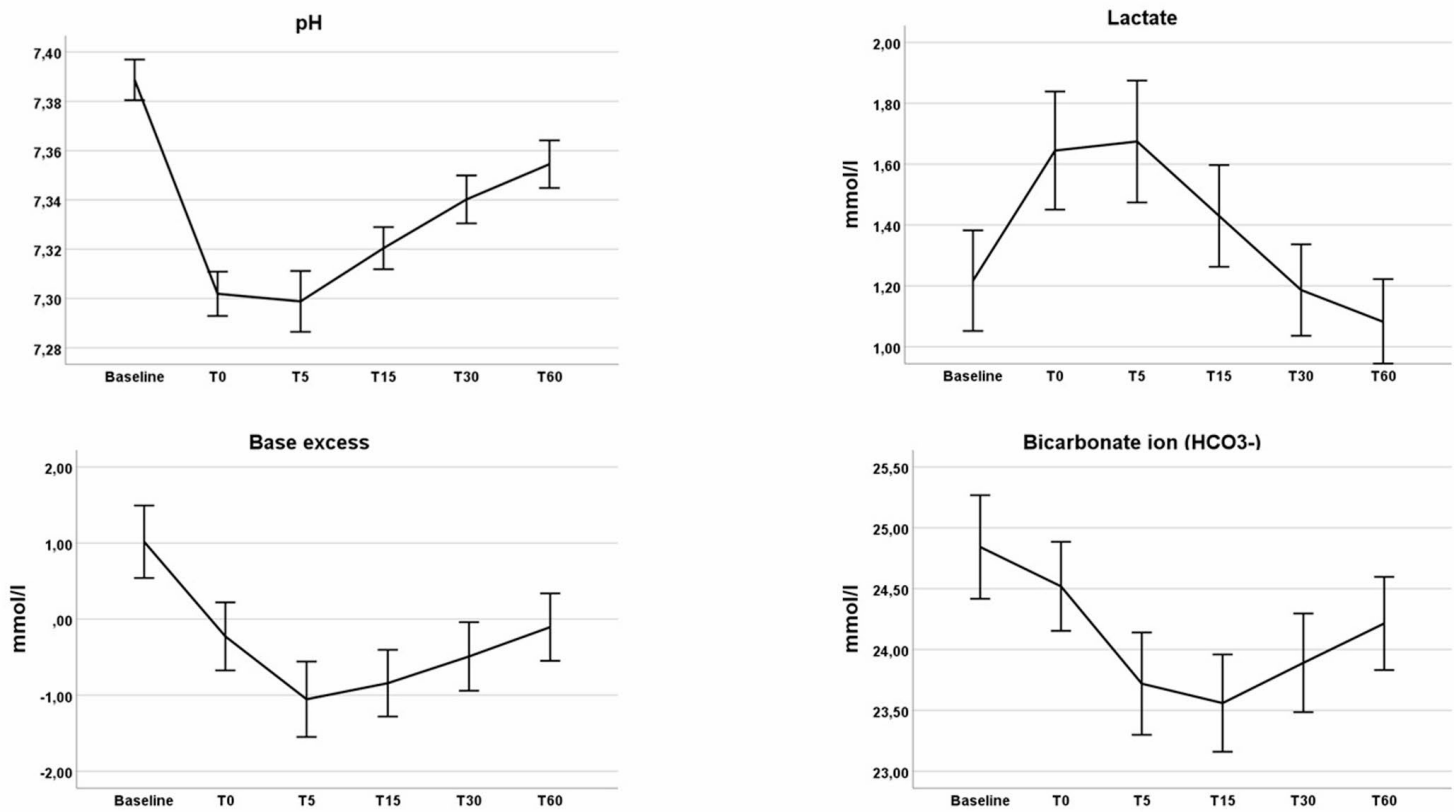
Course of pH, lactate, base excess, and bicarbonate ion concentration after ECT. T0, T5, and so forth indicate time points in minutes after ECT

This study showed a highly significant effect of ECT on pH, the associated standard bicarbonate, and the standardized base excess (*p* < 0.001 each for the cohort of all 47 sessions as well as the cohort of 19 independent ECT-series of the 19 patients included, Online Resource Table S1). While the initial pH value averaged 7.39 ± 0.03 (7.32–7.45), it decreased to a minimum of 7.3 ± 0.04 (7.17–7.36) 5 min after ECT, the base excess decreased from 1.0 ± 1.6 (−3–4.9) mmol/l to −1.0 ± 1.7 (−5.4–2.85) mmol/l 5 min after ECT, while standard bicarbonate decreased from 24.8 ± 1.45 (21.8–28.4) mmol/l to its mean trough value of 23.5 ± 1.36 (19.9–26.6) mmol/l 15 min after ECT (Fig. 1).

All parameters normalized or nearly normalized within one hour. The largest intra-individual drop in pH was 0.19 (from initial 7.36 to 7.17 5 min after ECT) and correlated with the largest intra-individual deviation in pvCO2 (see below). The largest decrease in base excess was 5.7 mmol/l (decrease from 0.3 to −5.4 mmol/l at 5 min) and correlated with the maximum intra-individual variation in standard bicarbonate of 4.4 mmol/l (decrease from 24.5 before to 20.1 mmol/l 5 min after ECT) as well as the maximum intra-individual variation in lactate. No sex difference was found for any of these variables (pH: F(1;17) = 1.34; *p* = 0.26; *n* = 19); base excess: (F(1;17) = 2.92; *p* = 0.11; *n* = 19); HCO3-: (F(1;17) = 3.8; *p* = 0.07; *n* = 19). Bonferroni-corrected post-hoc pairwise comparisons revealed significant (*p* < 0.05) differences from baseline to multiple time points for most parameters (Online Resource Tables S2 and S3).

**Fig. 2:**
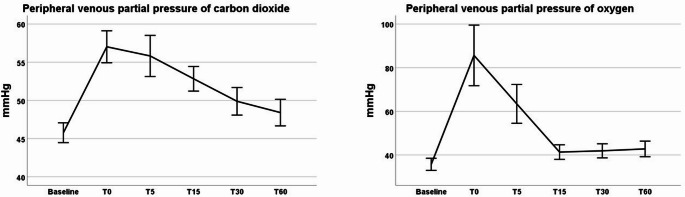
Course of venous CO2 and O2 partial pressure after ECT. T0, T5, and so forth indicate time points in minutes after ECT

Significant alterations after ECT were also recorded regarding plasma lactate, venous partial pressure of carbone dioxide, and venous partial pressure of oxygen (*p* < 0.001 each for the cohort of all 47 sessions as well as the cohort of 19 independent ECT-series of the 19 patients included, Online Resource Table S1). While the lactate value increased from 1.22 ± 0.56 (0.5–3.4) mmol/l to a maximum of 1.67 ± 0.68 (0.8–4.8) mmol/l 5 min after ECT, the maximum peak values of pvCO2 were 57 ± 7.1 (46–71) mmHg (from a baseline of 47 ± 4.4 (38–60) mmHg)) and 86 ± 47.3 (34–252) mmHg for pvO2 (baseline of 36 ± 9.5 (20–55) mmHg) (Fig. 2).

Lactate and blood gases normalised within the first hour in most cases (Online Resource Tables S2 and S3). The largest single deviations from baseline were 2.5 mmol/l (from 2.6 mmol/l before to 5.1 mmol/l immediately after ECT) for lactate, 33 mmHg (from 48 mmHg before to 81 mmHg 5 min after ECT) for pvCO2, and 210 mmHg (from 42 to 252 mmHg immediately after ECT) for O2.

For lactate, only two patients accounted for 22 of the 24 values above 2.2 mmol/l, which in three sessions were already elevated before ECT. Both theses patients suffered from type 2 diabetes and received metformine or sitagliptine, respectively. Lactate values were on average lower in women (F(1;17) = 6.4; *p* < 0.021; *n* = 19), whereas there was a trend for higher pvO2 values (F(1;17) = 4.1; *p* = 0.058; *n* = 19). Sex had no effect on pvCO2 (F(1;17) = 0.22; *p* = 0.65; *n* = 19). Results of Bonferroni-corrected post-hoc pairwise comparisons and absolute numbers of measurements within, below or above the respective reference ranges are shown in the Online Resource Tables S2, S3, and S4, respectively.

### Changes of electrolytes

Even though we found significant effects of ECT on sodium, chloride, and calcium levels, changes over time remained marginal. Sodium basically remained at 137 ± 2 (134–142) mmol/l, chloride at 106 ± 3 (101–110) mmol/l, and calcium between 1.21 ± 0.05 and 1.25 ± 0.05 (1.12–1.41) mmol/l. The largest deviations from baseline values were 6 mmol/l for sodium (141 to 135 mmol/l after 60 min), 4 mmol/l for chloride (110 to 106 mmol/l after 60 min) and 0.1 mmol/l for calcium (1.18 to 1.28 mmol/l immediately after ECT). After applying the Bonferroni correction, there were no significant differences for sodium and chloride levels after ECT. Differences between time points for calcium and absolute numbers of measurements within, below or above the respective reference ranges are shown in the Online Resource Tables S2, S3, and S4, respectively. Due to the unreliability of potassium values obtained by aspiration from a constant peripheral venous catheter, we excluded this electrolyte from the analysis.

### Glucose, hemoglobin, and hematocrit

**Fig. 3:**
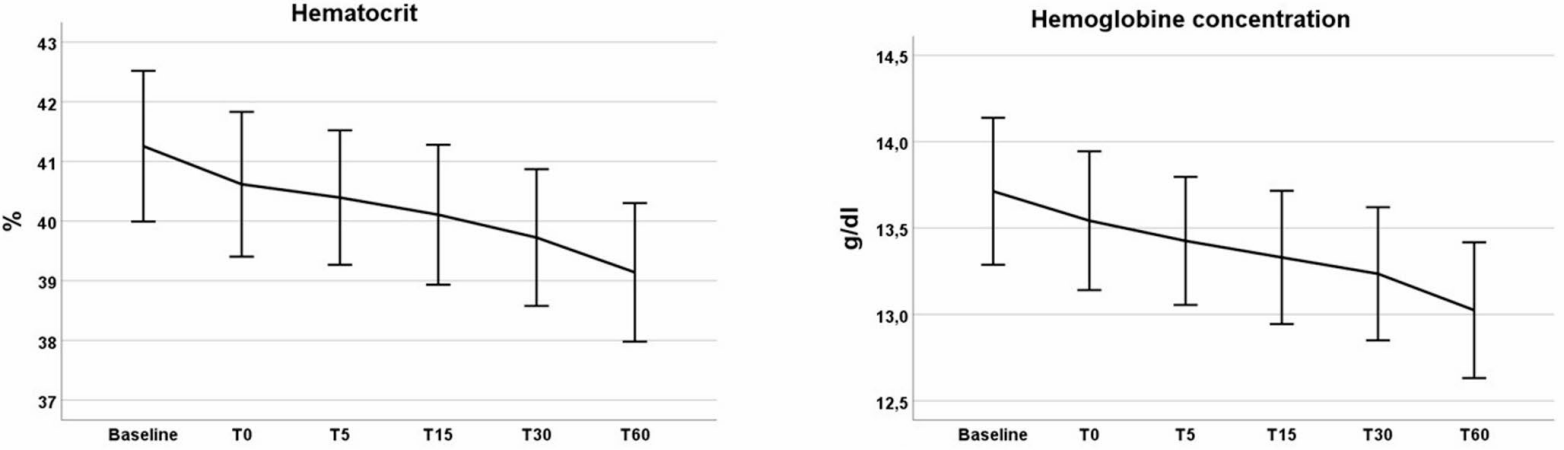
Course of hematocrit and Hb after ECT. T0, T5, and so forth indicate time points in minutes after ECT

Glucose, hemoglobin, and hematocrit were also significantly affected by ECT (*p* < 0.001 each for the cohort of all 47 sessions as well as the cohort of 19 independent ECT-series of the 19 patients included (glucose: *p* = 0.006, Online Resource Table S1). Taking into account all available measurements, glucose levels increased from an initial 106 ± 22 (86–226) mg/dl to a mean maximum of 112 ± 20 (85–224) mg/dl 15 min after ECT, while the post-ECT decrease in hemoglobin (from 13.7 ± 1.45 (11–15.6) g/dl to a minimum of 13 ± 1.34 (10.5–15) g/dl) and hematocrit (from 41.3 ± 4.3 (33–47) % to a minimum of 39.1 ± 4 (32–45) %) each reached its minimum 60 min after ECT (Fig. 3).

The largest deviation from baseline glucose was only 26 mg/dl (increase from 100 mg/dl before ECT to 126 mg/dl 15 min after). The most significant decrease in Hb was 2.4 g/dl (from 14.6 to 12.2 g/dl after 60 min) and in hematocrit was 5% (from 45 to 40% after 30 min). Blood glucose (F(1;17) = 5.5; *p* < 0.031; *n* = 19), and hemoglobin (F(1;17) = 5.0; *p* = 0.039; *n* = 19) were on average lower in women than in men acknowledging that the two patients with type 2 diabetes in our cohort were male. Differences between time points for glucose, hemoglobin and hematocrit and absolute numbers of measurements within, below or above the respective reference ranges are shown in the Online Resource Tables S3, S3, and S4, respectively.

We then determined the maximum deviation of the respective parameters from their baseline. The time-points after ECT, at which this deviation was maximal is to be found in the Online Resource Table S5.

Finally, we conducted correlation analyses between this difference and height, weight, age, as well as motor and EEG seizure duration. Significant positive correlations with patients’ height were detected for lactate. Only glucose values correlated positively with EEG-seizure duration (Table 2).

**Table 2:** Correlation analyses between the parameters obtained and potentially relevant factors

	Height	Weight	Age	t-mot.	t-EEG
pH	−0.05 (0.84)	−0.17 (0.51)	0.0.35 (0.14)	−0.11 (0.45)	−0.08 (0.62)
Lactate	**0.54 (0.02)**	0.2 (0.43)	0.17 (0.49)	0.06 (0.68)	−0.04 (0.8)
Base excess	−0.35 (0.16)	−0.09 (0.72)	−0.23 (0.34)	0.03 (0.83)	0.10 (0.51)
HCO3-	−0.19 (0.46)	−0.02 (0.94)	0.2 (0.42)	0.06 (0.68)	0.16 (0.29)
pvCO2	0.12 (0.66)	0.25 (0.33)	0.27 (0.27)	0.09 (0.54)	0.15 (0.31)
pvO2	−0.38 (0.12)	−0.38 (0.12)	−0.02 (0.93)	0.07 (0.63)	0.06 (0.71)
Sodium	−0.19 (0.45)	−0.13 (0.62)	−0.15 (0.55)	−0.15 (0.31)	−0.02 (0.88)
Chloride	−0.14 (0.59)	0.18 (0.47)	0.05 (0.84)	0.20 (0.18)	0.25 (0.09)
Calcium	−0.19 (0.45)	−0.05 (0.84)	−0.13 (0.6)	−0.01 (0.97)	0.07 (0.65)
Glucose	0.39 (0.11)	0.43 (0.08)	0.19 (0.44)	0.22 (0.14)	**0.34 (0.02)**
Hemoglobin	0.35 (0.16)	0.02 (0.95)	−0.05 (0.84)	−0.22 (0.14)	−0.14 (0.34)
Hematocrit	0.41 (0.09)	0.08 (0.77)	−0.06 (0.81)	−0.24 (0.11	−0.16 (0.29)

For height, weight and age correlation analysis was based on the biometric data of the 19 patients of the cohort and their respective first series. For EEG- and motor seizure duration all 47 sessions were included. Cells of the table provide correlation coefficients and respective p-values in brackets. Numbers in bold represent significant results.

## Discussion

In the present study, we investigated the course of laboratory parameters that can be determined by blood gas analysis within the first hour after ECT and possible associations with clinical ECT parameters. We documented significant, but short-lived and self-limiting changes in the acid-base status, while electrolytes basically remain stable after ECT. There were hardly any correlations of laboratory with biometric parameters or seizure duration. Our results confirm a high degree of safety of ECT with respect to effects on blood-homeostasis.

Based on the kinetics of pvCO2 and lactate the pH dropped from 7.39 ± 0.03 to 7.3 ± 0.04 within 5 min of ECT and then rose again during subsequent measurements (Figs. 1 and 2). As free H + ions are absorbed by the buffer bases, especially HCO3, the maximum decrease of the latter and the base excess 15 min after ECT reflects the kinetics of the metabolic counter-reaction to acidosis according to the Henderson-Hasselbalch equation. Therefore, this parameter reached its maximum deflection at the latest among the acid-base parameters. In a study in which comparable measurements were made in patients with sporadic epileptic seizures presenting to emergency physicians, bicarbonate levels were found to be on average 17 mmol/l within the first half hour (Bakes et al. 2011), which is significantly more pronounced than in our study. With reference to post-ECT measurements, different results were obtained by Szirmai et al. (Szirmai et al. 1975) who measured the pH in the internal jugular vein, and found only very mild alterations immediately after ECT. The fact that blood was taken from the internal jugular vein, which reflects brain metabolism, may have contributed to these rather different results. While the average increase in lactate after ECT was about 0.5 mmol/l much more significant lactate increases have been reported in historical studies from the early days of ECT. In these, lactate levels doubled with ECT when administering rather moderate doses of succinylcholine, and increased by a factor of five (Mitis et al. 1954) or six (Eiduson et al. 1953) when muscle relaxation was omitted. The potential importance of the succinylcholine dose was also observed in our cohort. The largest deviation of lactate from baseline (from 2.6 mmol/l before to 5.1 mmol/l immediately after ECT) coincided with a possibly too low succinylcholine dose of 80 mg in one male patient. When the same patient was re-treated with a succinylcholine dose of 120 mg, the lactate increased by only 0.5 mmol/l. Interestingly, in two sessions we observed a decrease in lactate immediately after ECT (from 3.1 to 2.7 and from 3.4 to 2.7 mmol/l, respectively), whereas lactate increased in all others. It is conceivable that in these two cases, lactate was preferentially used as an anaerobic energy source due to its high concentration. And, 22 of the 24 lactate values above 2.2 mmol/l in our cohort were accounted for by the same two patients with type 2 diabetes, who had elevated lactate and glucose values almost continuously - even prior to the ECT which conforms with the insulin-resistant metabolism. Metformin in one case and sitagliptine in the other - both of which can induce hyperlactatemia - may have contributed to this finding as well. In general, there was a significant but irrelevant increase of glucose levels of about 5% (from 106 to 112 mg/dl on average) which quickly returned to baseline. Similar changes have been documented after tonic-clonic seizures, where glucose levels increased by up to 14%, possibly due to catecholamine-induced glycolysis (Simon et al. 1984; Nass et al. 2019). We also found a positive correlation between glucose levels and EEG seizure duration. This may either be due to the more abundant substrate fueling seizure activity or correlate with glucose-related risk factors. Due to preoxygenation the pvO2 expectedly and in line with other studies increased from 36 ± 9.5 mmHg to 86 ± 47.3 mmHg immediately after ECT and proved to be the parameter that returned to pre-ECT levels most quickly (after only 15 min) (Szirmai et al. 1975; Swindells and Simpson 1987).

The sodium and chloride concentrations remained essentially unchanged by ECT which is consistent with older studies (Lafferty et al. 2001). Similarly, sporadic tonic-clonic seizures result in a small increase in serum sodium only (140 to 142 mmol/l on average), returning to baseline within two hours (Nass et al. 2019) or have no effect on chloride concentrations (Brivet et al. 1994; Li et al. 2017). Correspondingly, mild deviations of serum sodium within the reference range prior to an ECT session have no or clinically non-significant effects on the quality of ECT (Belz et al. 2020; Karl et al. 2024). Given the known negative relationship between calcium solubility and pH ionised calcium levels increased by 3% immediately after ECT (from 1.21 ± 0.05 to 1.25 ± 0.05 mmol/l) alongside acidification and then normalized, which is observed immediately after tonic-clonic seizures as well (Nass et al. 2019). Cytolysis or active transport processes e.g. sodium-calcium exchangers or active calcium efflux pumps may additionally have contributed to these alterations (Oceandy et al. 2006; Berrocal and Mata 2023). The 2% drop of the hematocrit and 0.5–1 g/dl decrease of the hemoglobin concentration during the first hour after ECT are due to hemodilution by intravenous volume substitution with a complete electrolyte solution, and basically in agreement with the results of others (Grathwohl et al. 1996; Chaturvedi et al. 2001).

Encouragingly, there were few safety concerns related to laboratory chemistry. Potentially safety-relevant changes occurred only in pH-associated values due to mild to moderate acidosis, with a minimum pH of 7.17 (pvCO2 of 82 mmHg) 5 min after ECT in our cohort. Overall, most pvCO2 values remained below 65 mmHg, with only two other values reaching 71 mmHg. Normalisation was rapid in all cases. Acidosis of any kind (metabolic, respiratory, or both) with a pH below 7.1–7.2 is generally regarded as an acute emergency, provided that its cause is not reversed in an expeditious manner. Fortunately, acidosis in ECT is self-limited. It is imperative that healthcare professionals are cognizant of the short-term effects associated with ECT, as acidosis can result in hyperkalemia and exert an influence on hemodynamics due to reduced inotropy, arterial vasodilation and an augmented risk of arrhythmias (Kraut and Madias 2010; Kimmoun et al. 2015). Consequently, patients diagnosed with a cardiomyopathy or hyperkalemia are predisposed to periprocedural complications, including acute cardiac failure or arrhythmias. In particular, during muscle relaxation, too low a dose may lead to increased muscle activity and thus more lactate release, whereas too high a dose may lead to prolonged respiratory depression and prolonged need for assisted ventilation. Whether short-term hyperoxemia up to 252 mmHg, which indicates adequate preoxygenation, has any relevant adverse effects is questionable. However, high doses of oxygen may have a vasoconstrictive effect on cerebral and coronary arteries (Damiani et al. 2018). With the exception of a slight positive correlation between glucose level and motor seizure duration, we found hardly any significant correlations between maximum deviations of laboratory parameters from baseline and biometric or seizure-related parameters. This is a little surprising, as one would have expected positive correlations between seizure duration and the blood concentration of acid substrates such as lactate and CO2. This may be due to the tendency of the induced seizures to be tailored in order to fulfil efficacy demands and decrease the burden of side-effects (i.e. surpassing a minimum seizure duration while avoiding long lasting seizures).

Limitations of this work include the use of mostly small caliber intravenous catheters (20 or 22 gauge) to obtain the respective blood samples. Additionally, simultaneous determination of arterial and venous blood would be theoretically superior to our method, but is difficult to justify under normal conditions. To better identify the source of the specific metabolic products, it would also be conceivable to determine cerebral oxygen saturation using the surrogate method of central venous oxygen saturation measurement.

In conclusion, this study summarizes the effects of an ECT-session on those laboratory parameters that can be measured by blood-gas-analyzers. Thereby, it provides profiles of immediate metabolic reactions accompanying an ECT-session. Importantly, significant alterations are generally short-lived, normalize spontaneously and are in line with the favorable safety-profile of ECT and its accompanying anesthesia.

## Data Availability

The raw data of the study can be provided upon request.

## References

[CR1] Aminoff MJ, Simon RP, Wiedemann E (1984) The hormonal responses to generalized tonic-clonic seizures. Brain 107(Pt 2):569–578. 10.1093/brain/107.2.569 6144354 10.1093/brain/107.2.569

[CR2] Bakes KM, Faragher J, Markovchick VJ et al (2011) The Denver seizure score: anion gap metabolic acidosis predicts generalized seizure. Am J Emerg Med 29:1097–1102. 10.1016/j.ajem.2010.07.014 20951531 10.1016/j.ajem.2010.07.014

[CR3] Belz M, Methfessel I, Spang M et al (2020) Overlooking the obvious? Influence of electrolyte concentrations on seizure quality parameters in electroconvulsive therapy. Eur Arch Psychiatry Clin Neurosci 270:263–269. 10.1007/s00406-019-01046-5 31317265 10.1007/s00406-019-01046-5

[CR4] Berrocal M, Mata AM (2023) The plasma membrane Ca2+-ATPase, a molecular target for Tau-induced cytosolic calcium dysregulation. Neuroscience 518:112–118. 10.1016/j.neuroscience.2022.04.016 35469971 10.1016/j.neuroscience.2022.04.016

[CR5] Brivet F, Bernardin M, Cherin P (1994) Hyperchloremic acidosis during grand mal seizure lactic acidosis. Intensive Care Med 20:27–31. 10.1007/BF02425050 8163754 10.1007/BF02425050

[CR6] Chaturvedi S, Chadda RK, Rusia U, Jain N (2001) Effect of electroconvulsive therapy on hematological parameters. Psychiatry Res 104:265–268. 10.1016/s0165-1781(01)00303-1 11728616 10.1016/s0165-1781(01)00303-1

[CR7] Damiani E, Donati A, Girardis M (2018) Oxygen in the critically ill: friend or foe? Curr Opin Anaesthesiol 31:129–135. 10.1097/ACO.0000000000000559 29334496 10.1097/ACO.0000000000000559

[CR8] Eiduson S, Kingsley GR, Portis RB, Wallace RD (1953) Effect of electroshock therapy on blood lactic acid and pyruvic acid. Proc Soc Exp Biol Med 84:365–367 13121041

[CR9] Grathwohl KW, Bruns BJ, LeBrun CJ (1996) Does hemodilution exist? Effects of saline infusion on hematologic parameters in euvolemic subjects. South Med J 89:51–55. 10.1097/00007611-199601000-00008 8545692 10.1097/00007611-199601000-00008

[CR10] Karl S, Sartorius A, Aksay SS (2024) No effect of serum electrolyte levels on electroconvulsive therapy seizure quality parameters. J ECT 40:47. 10.1097/YCT.0000000000000966 38411578 10.1097/YCT.0000000000000966

[CR11] Kimmoun A, Novy E, Auchet T et al (2015) Hemodynamic consequences of severe lactic acidosis in shock states: from bench to bedside. Crit Care 19:175. 10.1186/s13054-015-0896-7 25887061 10.1186/s13054-015-0896-7 PMC4391479

[CR12] Kraut JA, Madias NE (2010) Metabolic acidosis: pathophysiology, diagnosis and management. Nat Rev Nephrol 6:274–285. 10.1038/nrneph.2010.33 20308999 10.1038/nrneph.2010.33

[CR13] Lafferty JE, North CS, Spitznagel E, Isenberg K (2001) Laboratory screening prior to ECT. J ECT 17:158–165. 10.1097/00124509-200109000-00002 11528304 10.1097/00124509-200109000-00002

[CR14] Li Y, Matzka L, Maranda L, Weber D (2017) Anion gap can differentiate between psychogenic and epileptic seizures in the emergency setting. Epilepsia 58:e132–e135. 10.1111/epi.13840 28695610 10.1111/epi.13840

[CR15] Lipka K, Bülow H-H (2003) Lactic acidosis following convulsions. Acta Anaesthesiol Scand 47:616–618. 10.1034/j.1399-6576.2003.00115.x 12699523 10.1034/j.1399-6576.2003.00115.x

[CR16] Matz O, Heckelmann J, Zechbauer S et al (2018) Early postictal serum lactate concentrations are superior to serum creatine kinase concentrations in distinguishing generalized tonic-clonic seizures from syncopes. Intern Emerg Med 13:749–755. 10.1007/s11739-017-1745-2 28900842 10.1007/s11739-017-1745-2

[CR17] Meierkord H, Shorvon S, Lightman SL (1994) Plasma concentrations of prolactin, noradrenaline, vasopressin and oxytocin during and after a prolonged epileptic seizure. Acta Neurol Scand 90:73–77. 10.1111/j.1600-0404.1994.tb02682.x 7801741 10.1111/j.1600-0404.1994.tb02682.x

[CR27] Mishra A, Dave N (2013) Acute renal failure due to rhabdomyolysis following a seizure. J Family Med Prim Care 2(1):86–7. 10.4103/2249-4863.109962 10.4103/2249-4863.109962 PMC3894010 24479052

[CR18] Mitis ZK, Harris T, Nowinski WW (1954) The levels of peripheral blood lactic acid in psychiatric patients treated by EST, with or without the use of a muscle paralysant. Tex Rep Biol Med 12:305–312 13179051

[CR19] Nass RD, Zur B, Elger CE et al (2019) Acute metabolic effects of tonic-clonic seizures. Epilepsia Open 4:599–608. 10.1002/epi4.12364 31819916 10.1002/epi4.12364 PMC6885665

[CR20] Oceandy D, Buch MH, Cartwright EJ, Neyses L (2006) The emergence of plasma membrane calcium pump as a novel therapeutic target for heart disease. Mini Rev Med Chem 6:583–588. 10.2174/138955706776876177 16719833 10.2174/138955706776876177

[CR21] Orringer CE, Eustace JC, Wunsch CD, Gardner LB (1977) Natural history of lactic acidosis after grand-mal seizures. A model for the study of an anion-gap acidosis not associated with hyperkalemia. N Engl J Med 297:796–799. 10.1056/NEJM197710132971502 19702 10.1056/NEJM197710132971502

[CR26] Rao ML, Stefan H, Bauer J (1989) Epileptic but not psychogenic seizures are accompanied by simultaneous elevation of serum pituitary hormones and cortisol levels. Neuroendocrinology 49(1):33–9. 10.1159/000125088 10.1159/000125088 2716948

[CR30] Rodríguez-Villar S, Kraut JA, Arévalo-Serrano J, Sakka SG, Harris C, Awad I, et al. (2021) Acid-Base Working Group.. Systemic acidemia impairs cardiac function in critically Ill patients. EClinicalMedicine 37:100956. 10.1016/j.eclinm.2021.100956 10.1016/j.eclinm.2021.100956 PMC8255172 34258569

[CR22] Simon RP, Aminoff MJ, Benowitz NL (1984) Changes in plasma catecholamines after tonic-clonic seizures. Neurology 34:255–257. 10.1212/wnl.34.2.255 6538024 10.1212/wnl.34.2.255

[CR29] Stengl M, Ledvinova L, Chvojka J, Benes J, Jarkovska D, Holas J, et al. (2013) Effects of clinically relevant acute hypercapnic and metabolic acidosis on the cardiovascular system: an experimental porcine study. Crit Care 17(6):R303. 10.1186/cc13173 10.1186/cc13173 PMC4056780 24377654

[CR23] Swindells SR, Simpson KH (1987) Oxygen saturation during electroconvulsive therapy. Br J Psychiatry 150:695–697. 10.1192/bjp.150.5.695 3651707 10.1192/bjp.150.5.695

[CR24] Szirmai I, Boldizśar F, Fischer J (1975) Correlation between blood gases, glycolytic enzymes and EEG during electroconvulsive treatment in relaxation. Acta Psychiatr Scand 51:171–181. 10.1111/j.1600-0447.1975.tb00003.x 237409 10.1111/j.1600-0447.1975.tb00003.x

[CR25] Wagner KJ, Möllenberg O, Rentrop M et al (2005) Guide to anaesthetic selection for electroconvulsive therapy. CNS Drugs 19:745–758. 10.2165/00023210-200519090-00002 16142990 10.2165/00023210-200519090-00002

[CR28] Willert C, Spitzer C, Kusserow S, Runge U (2004) Serum neuron-specific enolase, prolactin, and creatine kinase after epileptic and psychogenic non-epileptic seizures. Acta Neurol Scand 109(5):318-23. 10.1046/j.1600-0404.2003.00232.x 10.1046/j.1600-0404.2003.00232.x 15080857

